# Supplementary material for: A bionic self-driven retinomorphic eye with ionogel photosynaptic retina
Source: Nat Commun. 2024 Apr 10;15:3086. doi: 10.1038/s41467-024-47374-6 (PMC11006927; doi:10.1038/s41467-024-47374-6)
Supplement: Supplementary file 1 — Supplementary Information [file 41467_2024_47374_MOESM1_ESM.pdf]

A bionic self-driven retinomorphic eye with ionogel photosynaptic retina

Authors: Xu Luo[1†], Chen Chen[1†], Zixi He[1†], Min Wang[1], Keyuan Pan[1], Xuemei Dong[1], Zifan Li[1], Bin Liu[1], Zicheng Zhang[1], Yueyue Wu[1], Chaoyi Ban, Rong Chen[1], Dengfeng Zhang[1], Kaili Wang[1], Qiye Wang[1], Junyue Li[1], Gang Lu[1], Juqing Liu[1*], Zhengdong Liu[1*], Wei Huang[1,2,3*]

Affiliations:

[1]Key Laboratory of Flexible Electronics (KLoFE) & Institute of Advanced Materials (IAM), School of Flexible Electronics (Future Technologies), Nanjing Tech University (NanjingTech), Nanjing, China.

[2]Frontiers Science Center for Flexible Electronics, Institute of Flexible Electronics (IFE), Northwestern Polytechnical University, Xi'an, China.

[3]State Key Laboratory of Organic Electronics and Information Displays, Nanjing University of Posts and Telecommunications, Nanjing, China.

*Corresponding author Email: iamjqliu@njtech.edu.cn; iamzdliu@njtech.edu.cn; iamwhuang@njtech.edu.cn.

†These authors contributed equally to this work.

Supplementary Table 1| The comparison of self-powered optical synapses.

Functional layers	PPF	Wavelength (nm)	Flexibility	Healability	Self-powered mechanism	Ref.
All-inorganic CsPbI3 nanowire	/	405-650	/	/	Photovoltaic	1
C_8-BTBT /F_{16}CuPc	136%	365-850	/	/	Photovoltaic	2
P(VDF-TrFE)/ $Cs_2AgBiBr_6$	152%	455	/	/	Photovoltaic	3
$MAPbI_3$:SiNCs	137%	375-808	/	/	Photovoltaic	4
Spiro-OMeTAD	130%	450-650	/	/	Photovoltaic	5
$Cs_2AgBiBr_6$/P(VDF-TrFE)	200%	445/660	/	/	Photovoltaic	6
Heterojunction ionogel	153%	365-970	√	√	Photothermo electronic	this work

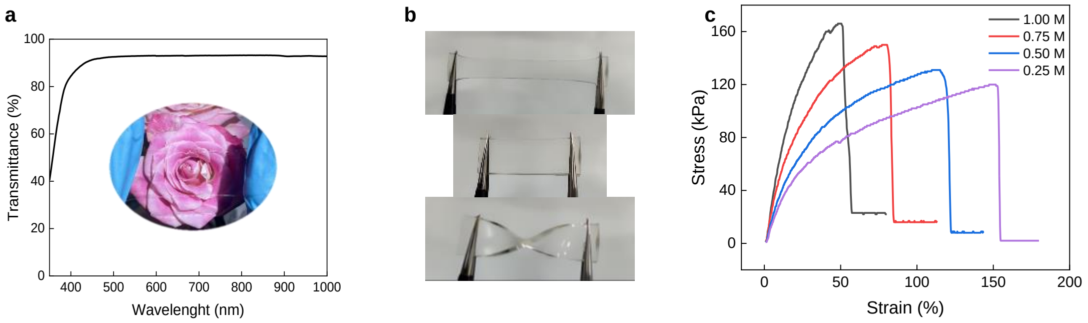

Supplementary Fig. 1| Optical and mechanical properties of synthetic ionogels. a, Transmittance of ionogel with an ionic salt concentration of 0.4 M. The insert figure is the photograph of ionogel with flower background. b, The photographs of ionogels in relaxed, twisted, and stretched states (relaxed state: 1 cm). c, Mechanical properties of ionogels with different ionic salt concentration. Tensile stress–strain curves show the fracture strength of the ionogels improved from 120 to 166 kPa, while strain at failure decreased from ~160 to 50% with the increase of doped ionic salt concentrations from 0.25 to 1.0 M.

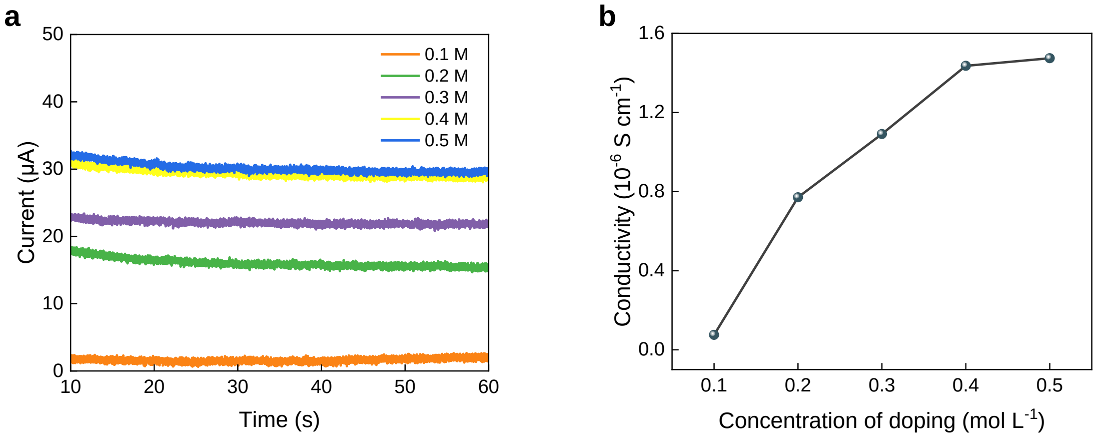

Supplementary Fig. 2| Electrical characterization of pure ionogels. a, Ionic conductivity of ionogels. The conductivity increases from 0.076 $\times 10^{-6}$ to 1.43 $\times 10^{-6}$ S cm^{-1} with the increase of doped ionic salt concentrations from 0.1 to 0.4 M. With further increase of the concentration to 0.5 M, the conductivity tends to saturation. b, The calculated electrical conductivity.

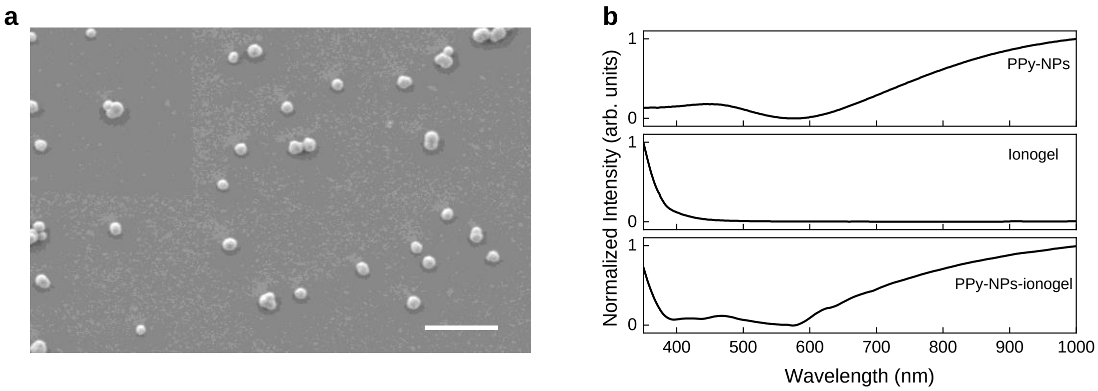

Supplementary Fig. 3| Morphology and optical spectrum characterizations. a, SEM image of polypyrrole nanoparticles (PPy-NPs). Scale bar, 1 μm. b, UV-vis-NIR spectra of the PPy-NPs, ionogel and PPy-NPs-ionogel. The pure ionogel exhibits a narrow absorption region at UV region while the PPy-NPs doped ionogel presents a broad absorption region from UV-vis-NIR region.

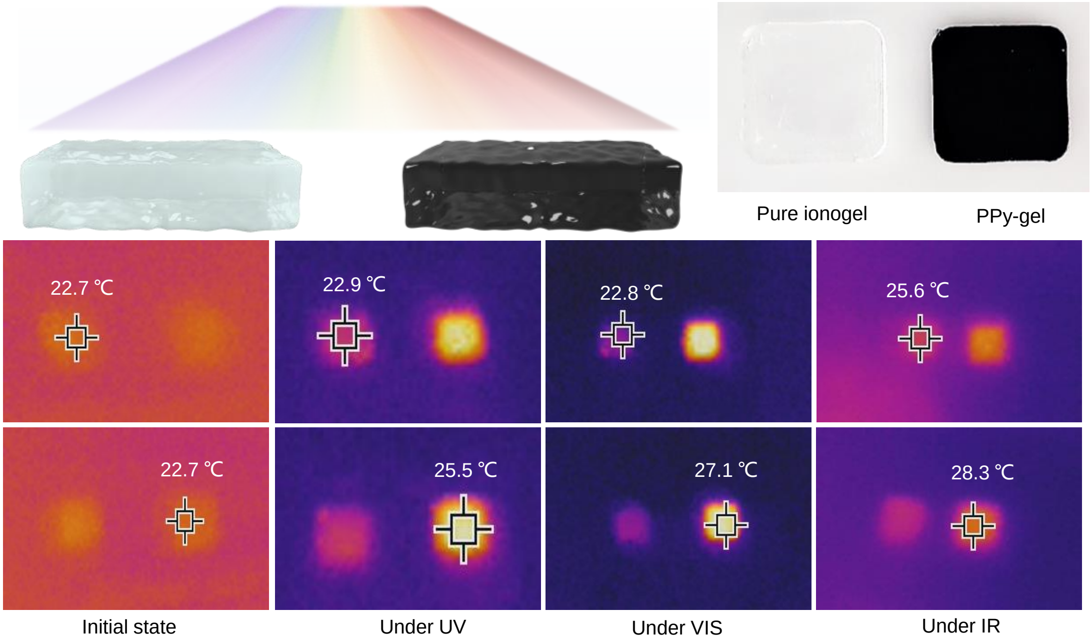

Supplementary Fig. 4| Photo–thermal conversion performance of ionogels with and without PPy-NPs doping. Under the light irradiation, both of the undoped and doped ionogels exhibit an increase of surface temperatures. Compared with the pure ionogel, the doped one exhibits a higher temperature under the same light irradiation.

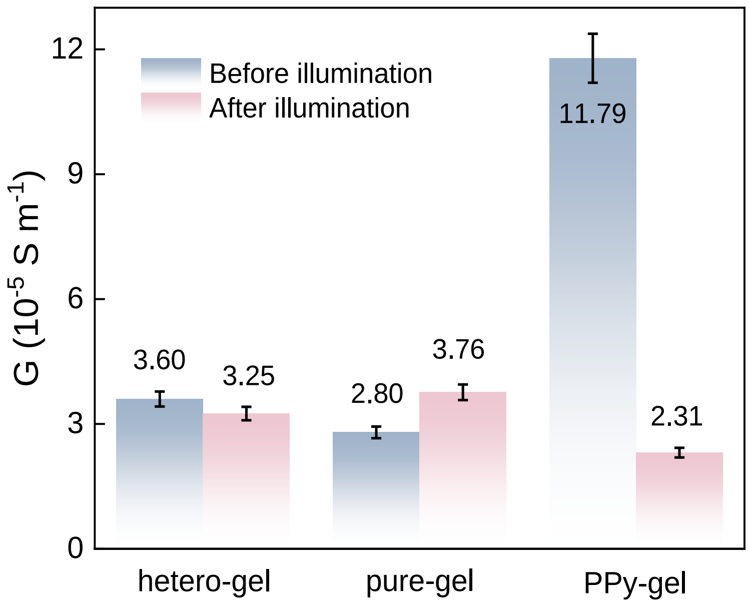

Supplementary Fig. 5| Ion migration under light exposure. To study the effect of thermodiffusion on ion flow, the ionic conductivity of each region in heterojunction were separately measured before and after light irradiation. Significant conductivity changes were observed, with a 9.48 $\times 10^{-5}$ S m^{-1} reduction in the doped region and a 0.96 $\times 10^{-5}$ S m^{-1} growth in the undoped region, due to ion drift from the hot (dopant) side to the cool (pure) side, which certified the light-driven ionic migration induced by photothermoelectric effect. Error bars represent standard deviations.

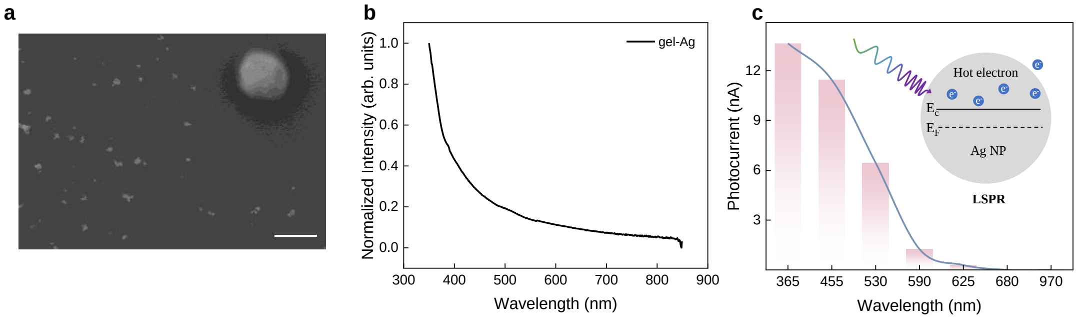

Supplementary Fig. 6| LSPR of metal nanoparticles. Localized surface plasmon resonance (LSPR) of silver nanoparticles can enhance the photon absorption capacity for photodetector light response. The conductive silver paste's nanoparticles exhibit exceptional UV-vis light absorption and generate a broadband photoelectric response through synergistic action. The photon energy absorbed by the silver nanoparticles can releases numerous electrons into the electrolyte, creating an electron-rich region on the ionogel side that attracts Li^+ migration (asymmetric light absorption). a, SEM image of silver paste depicting numerous nanoparticles. Scale bar, 2 μm. Particle diameter ≈ 300 nm. b, UV-vis-NIR spectra of the silver paste and c, wavelength and light response (optical power = 2 mW), exhibiting negligible photocurrent beyond 590 nm, as shown in the absorption spectrum in b.

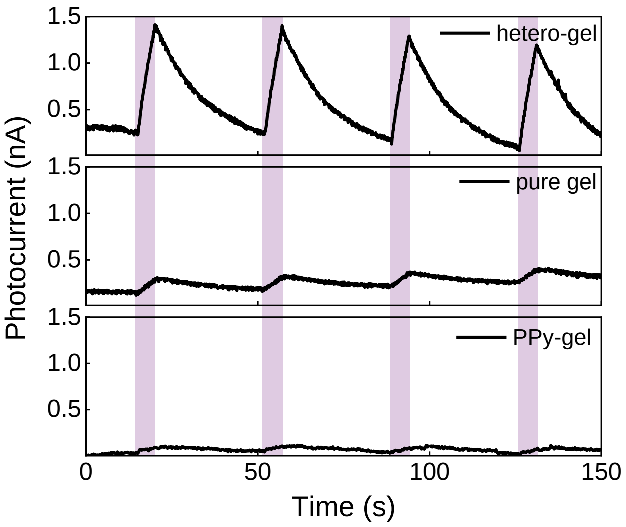

Supplementary Fig. 7| Comparison of photocurrent generated in heterojunction and non-heterojunction. Results show that the doping of PPy-NPs in heterojunction can enhance its photoresponse ability compared to that in non-heterojunctions.

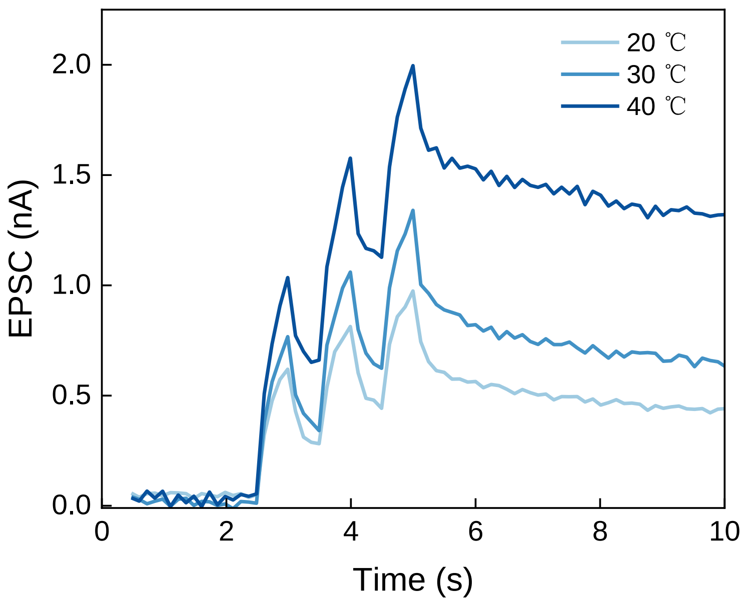

Supplementary Fig. 8| Temperature-dependent photocurrent properties. EPSC curves of the photoreceptor at different temperatures (365 nm, 14.4 μW mm^{-2}).

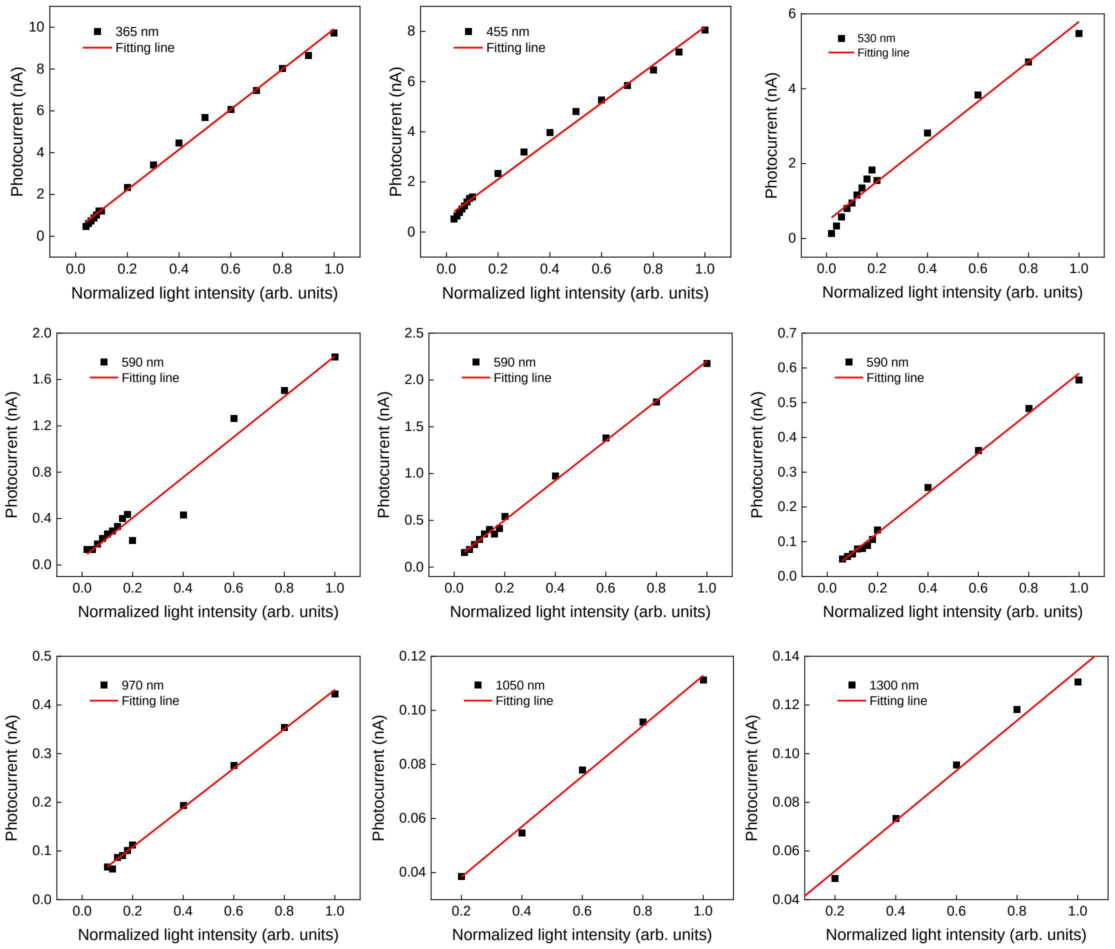

Supplementary Fig. 9| The dependence of responsivity and photocurrent on the illumination wavelength and intensity. The photoresponse ability of heterojunction shows a large dynamic range from 365 nm to 1300 nm. The lines represent linear fits to the illumination intensity. A quasi-linear power-law relationship can be utilized to accommodate the photocurrent under various light exposures.

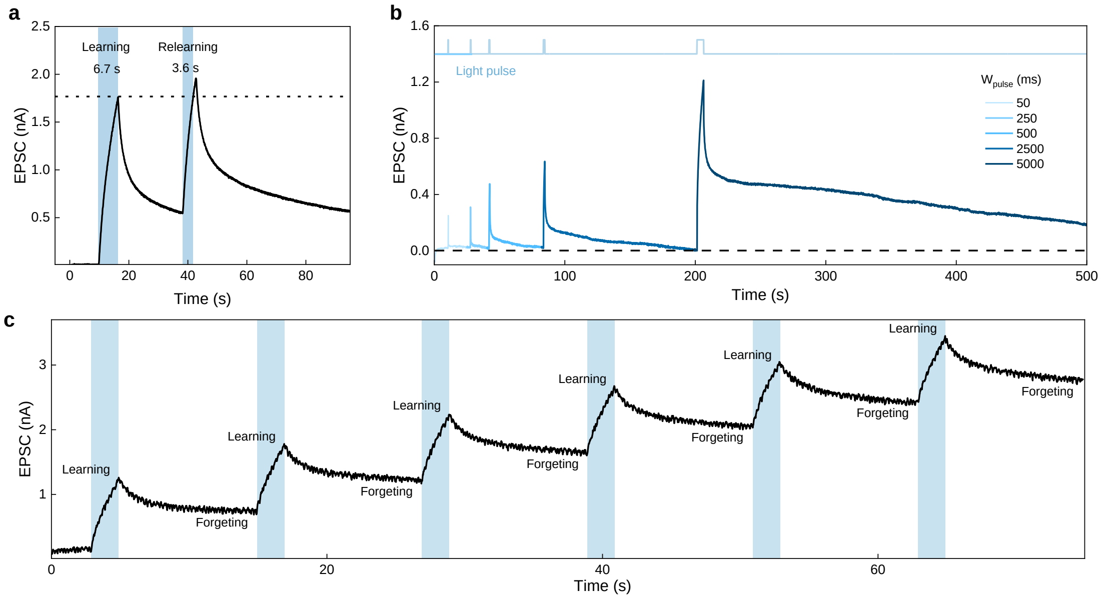

Supplementary Fig. 10| Neuroplasticity features of heterojunction photoreceptor. a, Transition from STM to LTM by lengthening the W_{pulse} under a fixed power (365 nm, 7.2 μW mm^{-2}). b, The postsynaptic photocurrent of photoreceptor under two consecutive optical pulses (365 nm, 7.2 μW mm^{-2}). c, The "learning-forgetting-relearning" experience behavior of the photoreceptor under successively optical pulses with a fixed pulse number (365 nm, 14.4 μW mm^{-2}).

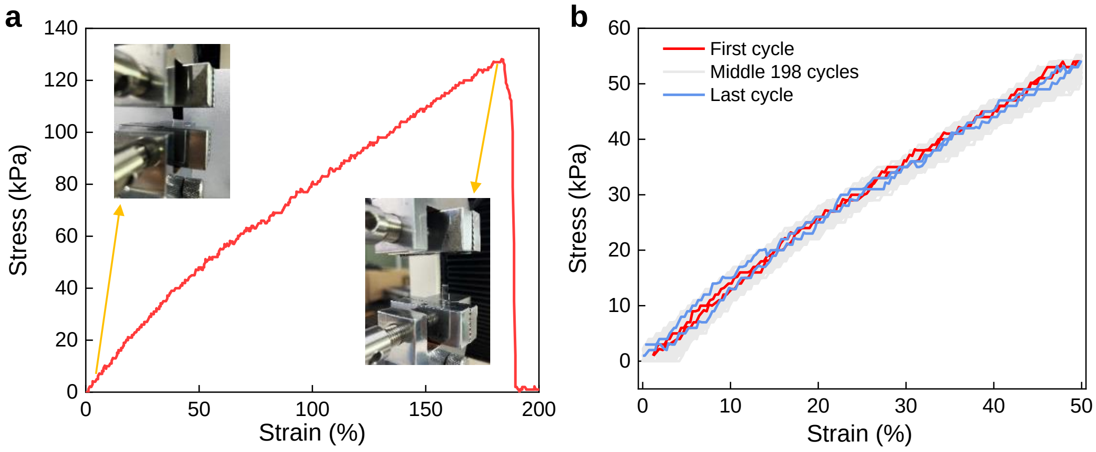

Supplementary Fig. 11| The mechanical property of heterojunction ionogel. a, Tensile stress-strain curve for heterojunction ionogel. Inset: Photograph of original state and maximum strain state. b, Cyclic stress-strain curves at a fixed strain of 50%. The device exhibits a stretching capability of approximately 180%, which effectively satisfies various application requirements. The stress-strain cycling curves of the device almost overlapped with its initial state even after undergoing 200 loadings at a stretch of 50%, indicating its reliable stability.

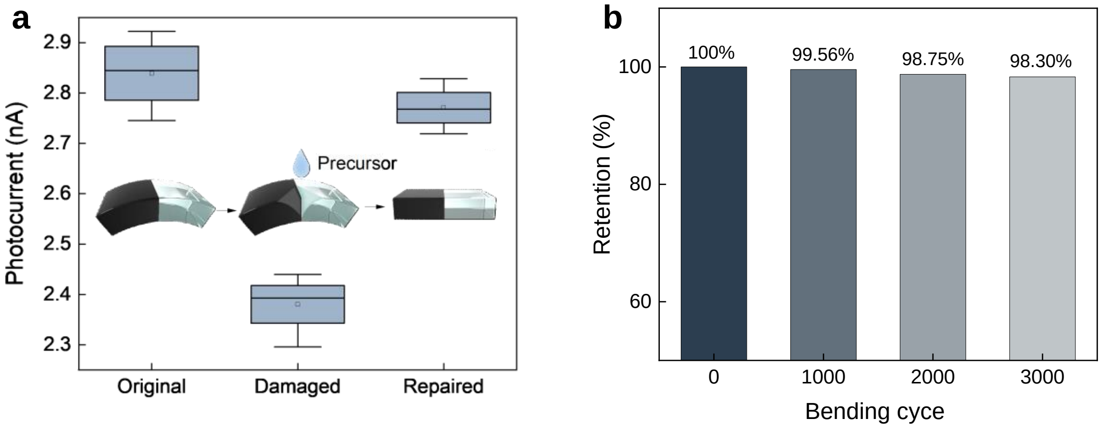

Supplementary Fig. 12| Electric-healing ability of retinal photoreceptor. a, The photocurrents of the same photoreceptor at different conditions: original, damaged, and repaired states. Error bars represent standard deviations. b, The photocurrent changes of the transplanted photoreceptor after bending for 1000, 2000, and 3000 times.

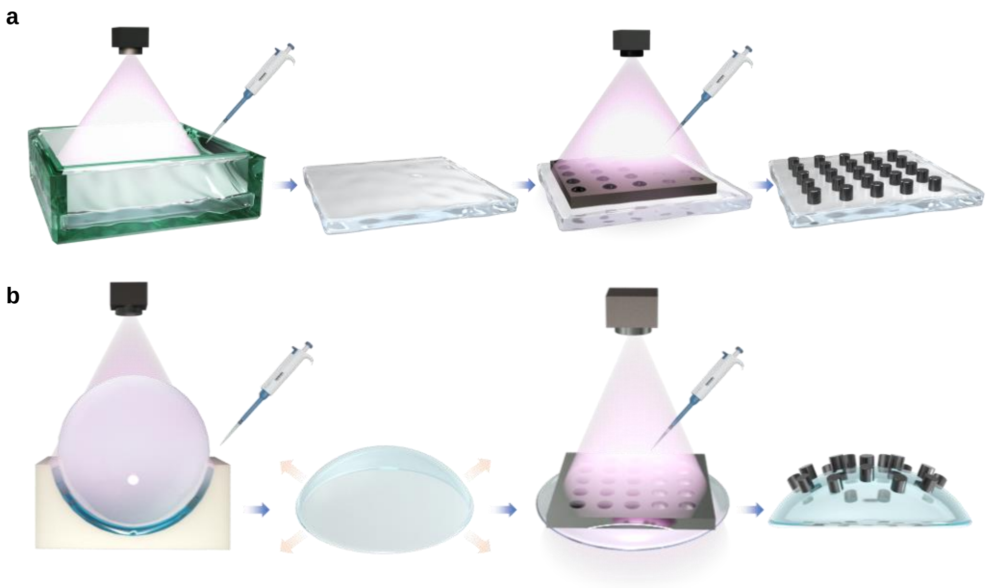

Supplementary Fig. 13| Schematic illustration of the fabrication of flat and hemispherical type retinal photoreceptors. a, The fabrication steps begin with the synthesis of flat pure ionogel in a square container. Transferring the ionogel onto a PET film and then it is covered with a mask plate. The precursor solution containing PPy-NPs is poured into the holes of the mask plate and then exposed to UV light. Removing the mask to obtain patterned PPy-NPs doped ionogel array. As a result, flat-type retina consisting of 25 photoreceptor cells is obtained. b, Similarly, the process begins with the preparation of a hemispherical ionogel formed between opposing glass sphere and concave poly(dimethylsiloxane). Stretching and paving the hemispherical ionogel onto the PET film. Then a mask plate is used to cover the surface of ionogel film for the preparation of PPy-NPs doped ionogel array. Hemispherical retinal photoreceptor is obtained after removing the mask plate.

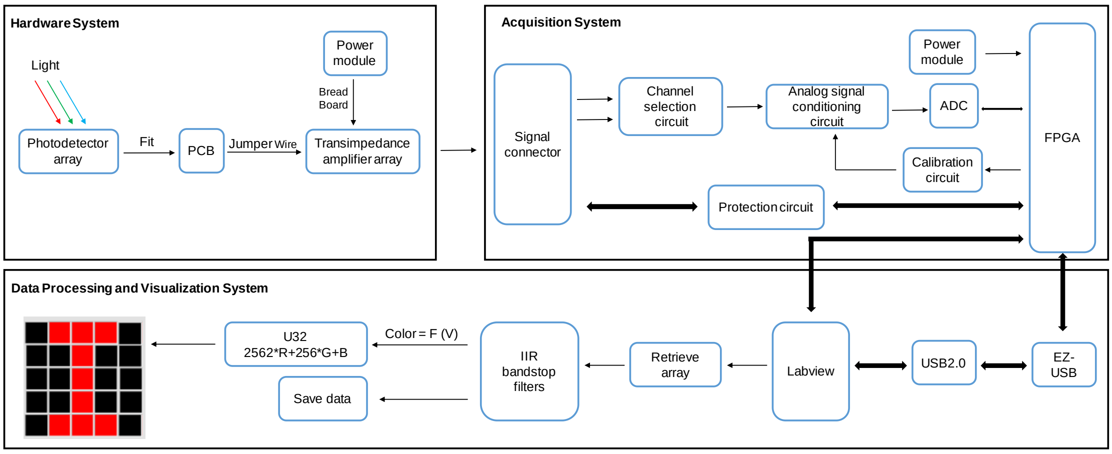

Supplementary Fig. 14| The construction of the automatic testing platform. The electrical signals generated by the photodetector array are conveyed to the trans-impedance amplifier (TIA) via a custom-designed printed circuit board (PCB). Following amplification, the signals are processed by the analog-to-digital converter (ADC) within the data acquisition card, which then uniformly collects and transmits the data to the Labview software on the computer (FPGA, field programmable gate array). Within the software, the data undergoes infinite impulse response digital filtering (IIR) to mitigate the interference of industrial frequency noise during transmission. Subsequently, the processed data is visualized in real-time through a self-designed visualization program on the computer.

Supplementary References

1 Long, Z. et al. A neuromorphic bionic eye with filter-free color vision using hemispherical perovskite nanowire array retina. *Nat. Commun.* **14**, 1972 (2023).

2 Hao, Z. et al. Retina-Inspired Self-Powered Artificial Optoelectronic Synapses with Selective Detection in Organic Asymmetric Heterojunctions. *Adv. Sci.* **9**, e2103494 (2022).

3 Lao, J. et al. Ultralow-Power Machine Vision with Self-Powered Sensor Reservoir. *Adv. Sci.* **9**, e2106092 (2022).

4 Huang, W. et al. Zero-power optoelectronic synaptic devices. *Nano Energy* **73**, 104790 (2020).

5 Sun, L. et al. An Artificial Reflex Arc That Perceives Afferent Visual and Tactile Information and Controls Efferent Muscular Actions. *Research* **11**, 9851843 (2022).

6 Lao, J. et al. Self-Powered and Humidity-Modulable Optoelectronic Synapse. *Adv. Mater. Technol.* **8**, 2201779 (2023).
